# The Importance of Social Engagement in the Development of an HIV Cure: A Systematic Review of Stakeholder Perspectives

**DOI:** 10.1007/s10461-023-04095-z

**Published:** 2023-06-17

**Authors:** Maaike A. J. Noorman, John B. F. de Wit, Tamika A. Marcos, Sarah E. Stutterheim, Kai J. Jonas, Chantal den Daas

**Affiliations:** 1 Department of Interdisciplinary Social Science, Utrecht University, PO Box 80140, 3508 TC Utrecht, The Netherlands; 2 Department of Work and Social Psychology, Maastricht University, Maastricht, The Netherlands; 3 Department of Health Promotion and Care and Public Health Research Institute, Maastricht University, Maastricht, The Netherlands; 4 Institute of Applied Health Sciences, Health Psychology Group, University of Aberdeen, Aberdeen, UK

**Keywords:** HIV cure, People with HIV, Meaningful involvement, Social engagement, Stakeholders

## Abstract

As research into the development of an HIV cure gains prominence, assessing the perspectives of stakeholders becomes imperative. It empowers stakeholders to determine priorities and influence research processes. We conducted a systematic review of the empirical literature on stakeholder perspectives. PubMed, Embase, Web of Science, and Scopus were searched for empirical, peer-reviewed articles, published before September 2022. Our analysis of 78 papers showed that stakeholders could be divided into three categories: people with HIV, key populations, and professionals. Following thematic synthesis, two main themes were distinguished: stakeholders’ perspectives on HIV cure research and stakeholders’ perspectives on HIV cure. Research on perspectives on HIV cure research showed that stakeholders’ hypothetical willingness to participate (WTP) in HIV cure research was relatively high, while actual WTP was found to be lower. Studies also identified associated (individual) characteristics of hypothetical WTP, as well as facilitators and barriers to hypothetical participation. Additionally, we reported research on experiences of actual HIV cure research participation. Our analysis of stakeholder perceptions of HIV cure showed that most stakeholders preferred a cure that could eliminate HIV and outlined positive associated impacts. Furthermore, we observed that most included studies were conducted among PWHIV, and in the Global North. To empower stakeholders, we recommend that future research include an even greater diversity of stakeholders and incorporate theories of behavior to further explore how stakeholders decide to meaningfully engage in every stage of HIV cure research.

**Supplementary Information:**

The online version contains supplementary material available at 10.1007/s10461-023-04095-z.

## Introduction

Since the first cases of AIDS were noted over 40 years ago, much progress has been made in HIV treatment [1, 2]. Effective treatment transformed HIV into a chronic, manageable condition as it prolongs life and reduces HIV transmission risk [2–4]. However, effective treatment is not curative and must be taken for life after diagnosis. The next frontier is the development of an HIV cure that could either eliminate HIV from the body (i.e., HIV eradication) or suppress HIV to the point that no further medication is needed (i.e., functional cure, remission, post-treatment control, or HIV suppression) [5]. The impact of an HIV cure for PWHIV will depend on the curative strategy developed, particularly whether or not a cure achieves long-term certainty about health, inability to transmit HIV, and a reduction in HIV-related stigma [2, 4]. Thus far, five individuals—Timothy Ray Brown, Adam Castillejo, the Düsseldorf patient, the New York patient, and the City of Hope patient—have been cured of HIV following stem cell transplantation [6-10]. However, this is not a suitable cure for most PWHIV as the procedure is complicated, invasive, and expensive. Creating a non-invasive and affordable curative strategy for all PWHIV is thus the current challenge [4]. The International AIDS Society (IAS) established the Global Cure Strategy initiative and identified five priority areas: understanding HIV reservoirs, mechanisms and models of virus control, therapeutic interventions, pediatric remission and cure, and social, behavioral, and ethical aspects of cure [11]. Currently, 335 trials and observational studies have been or are being conducted in search of a widely available HIV cure [12]. The majority of these trials are in phase I or II (N = 191) [12].

In addition to basic science, the IAS Global Cure Strategy recognizes the importance of social science in HIV cure research [11]. Social science research has the potential to complement basic science as it provides an understanding of HIV cure stakeholders’ values, beliefs, perceptions, and lived experiences [11, 13]. Including these perspectives empowers stakeholders [14], defined as anyone directly or indirectly associated with HIV cure, to determine priorities, identify concerns about HIV cure, influence HIV cure research practices, and influence the implementation processes [13].

The importance of social engagement, which is the process of actively involving stakeholders to define and influence issues concerning them [15], has been established in related domains such as HIV prevention and treatment [16]. For example, stakeholder and community engagement in efforts surrounding treatment-as-prevention (TasP), perinatal transmission prevention, and treatment of acute HIV infection, all helped to better translate research into practice [16]. Furthermore, history has shown that stakeholder consultations can effectively motivate health authorities, such as the World Health Organization (WHO), to revise guidelines and increase social justice, as was the case in the movement to make early ART initiation accessible to all PWHIV [16]. Likewise, early stakeholder engagement in HIV vaccine development has helped to manage expectations, mitigate failures, and increase acceptance of new trials [16]. Evidence demonstrating the value of stakeholder engagement is not limited to the field of HIV. In fact, Dubé et al. [17, 18] have argued that cancer research can provide substantial inspiration and can inform HIV cure research regarding important factors affecting participant engagement. Particularly, as HIV cure research modalities are heavily influenced by oncology research (e.g., use of anti-cancer drugs or cell and gene therapy) [18]. Drawing lessons from cancer research, we contend that HIV cure research should aim for a trusting relationship between the clinician-researcher and the patient-participant to ensure sensitive and ethical recruitment and decision-making processes.

Examples of where stakeholder engagement has been insufficient and inadequate also reinforce the importance of stakeholder engagement. For example, the failure to adequately engage stakeholders in pre-exposure prophylaxis (PrEP) research caused early termination or delayed implementation of several PrEP trials and may explain some of the current PrEP implementation difficulties [16]. In short, effective stakeholder engagement in HIV cure research is imperative, not only for the translation of basic science findings to the clinic, and, accordingly, participation in clinical trials but also because working together with stakeholders is ethical and just [14]. It redresses power imbalances, creates accountability, and reduces inequality between researchers and PWHIV [14].

Given the advances in basic science and the importance of social engagement in the field of HIV cure research, we conducted a systematic review that comprehensively summarizes the existing empirical literature on stakeholder perspectives in the field of HIV cure research and proposes important areas for future research on social engagement in HIV cure research.

## Methods

This systematic review was designed, conducted, and reported in accordance with the Preferred Reporting Items for Systematic Reviews (PRISMA) statement [19]. The review protocol is registered on PROSPERO, the International Prospective Register of Systematic Reviews (Registration Number CRD42020190942).

### Search Strategy

Four databases were searched for empirical literature on stakeholders’ perspectives on HIV cure: PubMed, EMBASE, Web of Science, and Scopus. The search terms included three key terms: stakeholders of HIV cure, the field of HIV cure research, and stakeholder views. These key terms and their variations were combined with the main Boolean operators “OR” and “AND” into one comprehensive search string (Supplementary Table I).

**Fig. 1:**
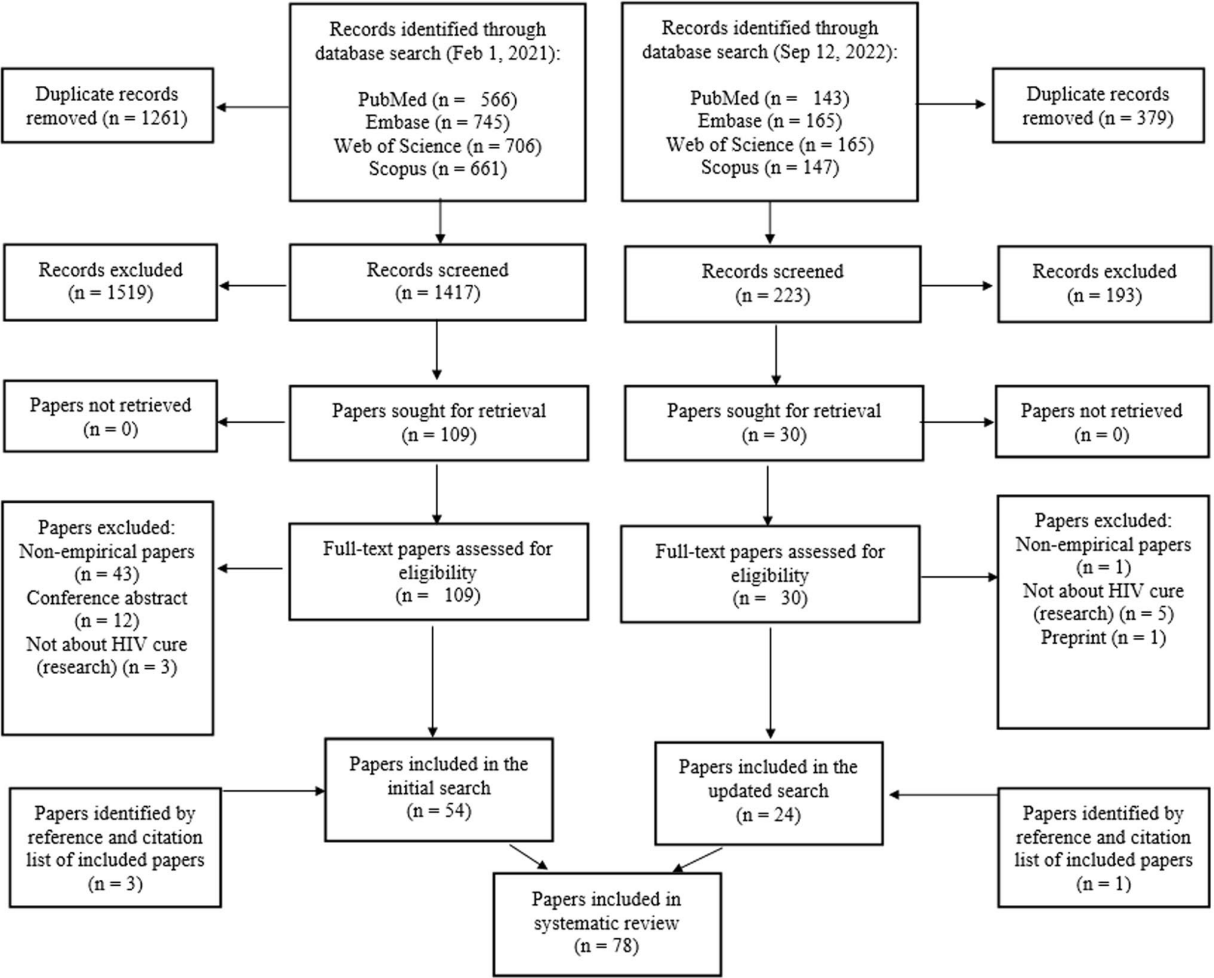
Flowchart of selection of papers for inclusion in the study

We conducted the initial search on 1 February 2021 and updated the search on 12 September 2022 (Fig. 1). Titles and abstracts of the initial search were screened by two independent reviewers (MAJN and TAM). The two reviewers obtained a high level of agreement (98.94%). Differences were resolved through discussion and, as necessary a third researcher (CdD) was consulted. As agreement was very high in the initial search, one reviewer (MAJN) updated the search. The other two researchers were consulted in the update if any uncertainty arose regarding the eligibility of a paper.

### Eligibility Criteria

Only peer-reviewed papers, written in English, reporting empirical findings, and published before September 12th, 2022, were eligible. Specific eligibility criteria were developed in line with the PICOTS (Population, Investigated condition, Comparison, Outcome, Timing, Study type) framework. Papers were eligible for inclusion when the population included HIV cure stakeholders which encompasses anyone directly or indirectly associated with HIV cure. Papers had to be affiliated with the field of HIV cure research and had to report on outcomes of participants who had completed a measure (e.g., questionnaire or survey) or provided an account (e.g., interview, focus group) of their perspectives. Study types included were qualitative, quantitative, or mixed analytic methods. Literature reviews, letters, opinion papers, and papers that did not report original data were excluded. Papers only reporting on clinical findings were also excluded. In total, 78 papers were included (Fig. 1).

### Data Extraction

Initial data extraction was performed by MAJN and verified by TAM. One researcher (MAJN) extracted the data from the updated search. For all papers, the following data were extracted: reference, location of the study, study design, sample size, type of stakeholders, and main theme(s) (Supplementary Table 2). For quantitative research papers, outcome data including descriptive and inferential statistics were extracted for measures of stakeholder views. Details on the measurement instruments were also recorded. For qualitative research papers, themes, categories, theories, and models describing stakeholder views were extracted. For mixed-method research papers, both outcomes of statistical measurements and qualitative findings representing stakeholder views were extracted.

### Quality Assessment and Risk of Bias

The initial appraisal of methodological quality and risk of bias of the included papers was undertaken by one researcher (MAJN) and verified by another (TAM). Quality was assessed through three standardized quality appraisal checklists designed by the National Institute for Health and Care Excellence (NICE) [20]. Quantitative experimental studies and observational studies received a methodological quality rating for internal and external validity. Qualitative studies received an overall assessment [20]. Based on the average scoring, papers could receive a high score (++), a moderate score (+), or a low score (−). A high score indicated that all or most of the criteria items were adequately described and met. Moderate scores meant that some of the checklist criteria were adequately described and met, but not all. Yet, the quality of studies was high enough that conclusions of the results were unlikely to have been affected. Low scores were given when few or none of the checklist criteria were fulfilled and conclusions were likely to have been affected [20].

### Data Analysis

Frequencies and percentages were calculated for the year of publication, study location, data collection method, sample size, type of stakeholder, and themes discussed among the different types of stakeholders.

The extracted data were analyzed through the three stages of thematic synthesis: coding, developing descriptive themes, and the generation of analytical themes [21]. Findings from the included articles were inductively coded to identify descriptive themes. Coding was carried out by one researcher (MAJN) and reviewed by another (TAM). Following this, analytical themes were distinguished, ordered, and discussed among the research team to determine the main themes and key messages.

## Results

Social science research on HIV cure is growing, with the number of papers published increasing in recent years (Table 1). Most papers presented studies conducted in the United States (N = 39, 50.00%, US). More than half of the studies reported a qualitative (N = 46, 58.97%) design with a sample size of fifty or fewer participants (N = 46, 58.97%).

**Table 1:** Characteristics of the included papers (N = 78)

Characteristics	Frequency	%
Year of publication		
2015–2016	6	7.69
2017–2018	18	23.08
2019–2020	29	37.18
2021–2022	25	32.05
Geographical setting		
United States	39	50.00
Australia	5	6.41
China	5	6.41
France	5	6.41
South Africa	5	6.41
Multi-country	6	7.69
Other countries < 5^a^	13	16.67
Study type		
Qualitative	46	58.97
Quantitative	25	32.05
Mixed methods	7	8.97
Sample size		
≤ 50	46	58.97
51 ≤ 250	16	20.51
251 ≤ 500	11	14.10
** > **500	5	6.41
Type of stakeholder^b^		
PWHIV	66	84.62
Key populations	23	29.49
Professionals	31	39.74

*PWHIV* people with HIV

^a^Other countries include: Thailand (N = 4), The Netherlands (N = 3), Belgium (N = 1), Brazil (N = 1), Canada (N = 1) Ghana (N = 1), Hong Kong (N = 1) and Switzerland (N = 1)

^b^Total frequencies exceed N = 78 and total percentage exceeds 100% because several papers included multiple stakeholders

According to quality appraisal, (Supplementary Table 3), there was little variation in the quality of papers. The majority of papers received a moderate score, meaning that some but not all criteria were adequately described and met. Nevertheless, even though not all criteria were adequately described and met, the quality of studies was high enough that the conclusions of results were unlikely to have been affected. Papers generally scored well on criteria related to recruitment and analyses. Lower scores were often given for checklist criteria assessing outcome measures. Criteria related to outcome variables scored lower as most data were self-reported and were often collected on non-validated scales or questionnaires. In addition, we observed that most papers took an explorative approach, frequently guided by principles of meaningful engagement of people with HIV/AIDS, community engagement, and research ethics principles. While these conceptual perspectives provide robust foundations and frameworks, we note the limited use of more specific theories of behavior, which can provide additional insight into the proximal factors that may shape behavior and mediate broader social and structural influences.

Not accounting for potential overlap between study participants, a total of 12,325 participants were sampled across all included papers. More than 20 different types of stakeholders were identified. For the purpose of our analyses, these stakeholder types were categorized into three groups based on similar recruitment criteria and participant information provided by the authors of the included papers. These three stakeholder groups are PWHIV (N = 66, 84.62%), key populations (N = 23, 29.49%), and professionals (N = 31, 39.74%). Key populations encompass people affected by HIV who were not explicitly described as people living with HIV. Their HIV-status was reported as HIV negative or was not reported. Key population participants were also not described as being employed in the HIV sector, including in relation to HIV cure researchers. Examples of key populations include men who have sex with men, people who inject drugs, transgender people, next of kin, and HIV activists. Professionals comprised all stakeholders who work in HIV-related fields, including researchers, healthcare professionals, social workers, policy makers, bioethicists, or pharmaceutical industry representatives. We recognize that these three stakeholder groups are not necessarily mutually exclusive and can overlap. However, the distinctions were made based on the recruitment criteria and participant information provided by the authors of included papers.

Two main themes were distinguished in the papers: perspectives on HIV cure research (N = 66, 84.62%) and perspectives on HIV cure (N = 27, 34.61%) (Supplementary Table 4). Sub-themes of perspectives on HIV cure research included: meaning of HIV cure research, willingness to participate (WTP), perceived facilitators and barriers for participation and conducting HIV cure research, engagement of stakeholders, and experiences with HIV cure research. Sub-themes of perspectives on HIV cure were awareness, meaning attributed to HIV cure, perceived impact, and stance on an HIV cure.

### Perspectives on HIV Cure Research

#### Perspectives of People with HIV

Several studies, reporting on both qualitative and quantitative results, found that most PWHIV were supportive of HIV cure research and believed that it was important, as it could *mean* scientific and health advancements [27-32]. Findings of primarily cross-sectional survey research showed that PWHIV’s hypothetical *WTP* in different forms of HIV cure research was relatively high, with most studies reporting that at least 50% of the research population is willing to participate (Table 2) [23, 24, 26, 29–42]. It is noteworthy that, in studies where HIV cure research was more extensively defined with riskier procedures, WTP was lower [24, 34, 38, 39, 42–45]. In addition, one cross-sectional survey study also compared actual participation rates to hypothetical WTP judgment in the Netherlands and found that hypothetical WTP was significantly higher among PWHIV [31].

**Table 2:** Operationalization and findings regarding people with HIV’s willingness to participate (WTP) in HIV cure research (N = 21)

Reference Country	Study design (data collection)	WTP measure	Outcome	Participant characteristics related to WTP
Arnold, Evans [33] United States	Quantitative (survey)	WTP in ATI (5-point likert scale)	34% very willing or willing 34% somewhat willing	*Personal benefit, social benefit, and scientific benefit:* all had a positive relationship with WTP *Viral load:* persons with unsuppressed (> 50/DK) viral loads had a lower WTP *ART regimens:* persons with 2 or more ART regimens had a higher WTP *Race/ethnicity:* black respondents reported lower WTP. Latino respondents reported higher WTP *Age:* 60 years or older had a higher WTP
Bonney, Lamptey [34] Ghana	Quantitative (survey)	Are you interested in participating in HIV cure research?	100% interested	*Age*: participants of under 40 years had a lower WTP in ATI. Participants above 55 years had a higher WTP in apheresis studies *Education:* Higher education was associated with more WTP in ATI. Participants with middle school education or higher education, were twice as likely to donate a quarter-punt of blood
		Are you WTP in:		
		1. surveys and interviews	1. 97% WTP	
		2. donate blood (quarter-pint)	2. 96% WTP (46% WTP)	
		3. add more medications to current ART	3. 70% WTP	
		4. undergo phase II or phase III clinical trial	4. 44% WTP	
		5. latency reactivation	5. 49% WTP	
		6. pheresis procedure	6. 23% WTP	
		7. ATI	7. 13% WTP	
Dubé, Evans [43] United States	Qualitative (interview)	WTP in ATI (open question)	Most PWHIV perceived ATIs as too risky for them personally	N.A.
Dube, Evans [35] United States	Quantitative (survey)	WTP in 14 types of specific HIV cure-related studies *Ranging from survey research to allogeneic transplant of stem cells*	Over 50% WTP in all 14 types of HIV cure studies 68% indicated that they were somewhat willing to stop treatment as part of HIV cure research	*Gender*: Females had a lower WTP in studies involving latency-reversing agents, gene modification, autologous stem cell transplant, and therapeutic vaccines *Race/ethnicity*: African Americans had a lower WTP in studies involving latency-reversing agents, gene modification, autologous stem cell transplants, therapeutic vaccines, and antibodies or molecules. Hispanics had a lower WTP in studies involving autologous stem cell transplants, therapeutic vaccines, treatment intensification, and antibodies or molecules *Income*: individuals in the lowest income bracket had a lower WTP in nearly all studies *Health*: individuals in poorer health had a higher WTP in studies involving latency-reversing agents and allogeneic stem cell transplants than healthier people *Diagnosis*: recently diagnosed individuals had a higher WTP in all studies *Perceptions of benefits* were positively correlated with WTP *Perceptions of risks* were negatively correlated with WTP
Dubé, Simoni [44] United States	Qualitatively (focus group)	WTP in cell and gene therapy	The procedure was regarded as risky which caused participants to not want to participate, only a few indicated that they were willing	N.A
Fiorentino, Protière [36] France	Quantitative (survey)	WTP in clinical trial based on your preferred HIV cure strategy (5-point Likert scale)	43% would definitely participate in an HIV-related clinical trial 38% would perhaps participate in an HIV-related clinical trial	*Self-identification as an HIV activist:* was positively associated with WTP *Self-confident as a PWHIV:* was positively associated with WTP
Gilles, Lesage [45] Switzerland	Qualitative (interview)	WTP in cell and gene therapy trial	6/15 participants were willing to participate	*Research experience:* Participants who had no research experience had a higher WTP
Kratka, Ubel [50] United States	Qualitatively (interview)	Willingness to risk death for a cure (participants rated this between 0 and 100%)	Median 30%, interquartile range: < 1%–60%	
Kwan, Chan (40) Hong Kong	Quantitative (survey)	Willingness to join an HIV functional cure study (6-point Likert scale)	93% would consider joining	*STI diagnosis in the past year* was associated with higher WTP *Taking advice from healthcare professionals* was associated with a higher WTP
Lessard, Dube [23] Canada	Mixed methods (survey and interview)	Participation in HIV cure research at the end of life	81.1% said yes	N.A.
		Participation in HIV biobanking	81.1% said yes	
		A research autopsy is being performed	75.7% said yes	
Murray, Kratka [24] United States	Quantitatively (survey)	Willingness to risk death for a cure		The following variables were positively associated with maximum risk of death Having higher *financial status* Rating current *health related quality of life* less high Expecting *side effects* from their medications in 20 years Expecting *no response from current HIV medication* in 20 years
		1. If there was 1 in 100 chance you would die, would you take the treatment?	1. 73% definitely or probably would	
		2. If there was 99 in 100 chance you would die, would you take the treatment?	2. 26% definitely or probably would	
		3. participants were asked to give the largest chance of death between 0 and 100% that would be acceptable	3. Median 10% Interquartile range: 15%–75%	
Peay, Rennie [41] Thailand	Quantitative (survey)	Participate in ATI time to rebound Participate in ATI sustained viremia design (strongly agree-agree-disagree-strongly disagree)	88% willing to join the time to rebound trail 68% were willing to join the sustained viremia trial	For *both* trials: *Making decisions by yourself* was associated with higher WTP *Agreeing with the statement that participating would be too burdensome:* was associated with lower WTP For t*ime to rebound* trail*:* *Those who agreed they would feel safe due to trail monitoring* had higher WTP *Those who agreed that they would be excited about joining* had higher WTP For *sustained viremia* trail: *Those who agreed that it would be easy to decide whether to join* had a higher WTP
Poteat, Aqil [29] United States	Qualitative (interviews)	Participate in HIV cure related research? (open-ended question)	Almost all participants were willing to participate in treatment interruptions; however, several had certain conditions such as receiving monetary compensation, being closely monitored, and interrupting ART for a very limited time	N.A.
Power, Westle [30] Australia	Qualitative (interviews)	HIV cure research (open-ended question)	18/20 (90%) participants would consider participating in HIV cure research	N.A.
Prakash, Gianella [37] United States	Quantitative (survey)	Willingness to shorten lifespan for end-of-life HIV cure research	69.2% were willing to shorten their lifespans for the sake of end-of-life research	N.A
Prins, Paulus [31] Netherlands	Quantitative (survey)	Hypothetical WTP versus actual WTP	67% was hypothetically WTP 43% was actually WTP	N.A.
Protière, Spire [32] France	Mixed method (Q-sort exercise)	WTP in HIV Cure-related clinical trials (Q-sort exercise)	63% WTP 34% yes, perhaps	N.A.
Sauceda, Dubé [26] United States	Quantitative (experiment)	WTP to participate in HIV cure clinical trial based on the informed consent form	59% WTP (no significant difference because of the informed consent form)	*Positive affective evaluation in informed consent forms* was associated with a higher WTP
Simmons, Kall [38] Multi-country	Quantitative (survey)	WTP in HIV cure research	95% were interested in participating in cure studies	Factors associated with WTP with substantial risk: *CD4 count:* 201–350 cells/μL compared with a current CD4 count of ≥ 350 cells/μL had a higher WTP *Nationality:* USA‐born compared with UK‐born had a higher WTP *Knowledge:* little or no knowledge about HIV or HIV treatments compared with excellent/good knowledge had lower WTP *Age:* Those aged ≥ 65 years compared with those aged 45–64 years had lower WTP No factor was associated with ATI
		WTP in HIV cure research with substantial risk defined as severe/moderate side effects without personal benefit	59% were willing to accept substantial risks	
		WTP in ATI	62% were willing to have treatment interruption	
van Paassen, Dijkstra [42] Netherlands	Qualitative (interviews)	Scenario 1 brief ATI	11/20 participants were willing to participate	N.A.
		Scenario 2 extended ATI	1/20 participants were willing to participate	
Wozniak, Cerqueira [39] Brazil	Quantitative (survey)	WTP in 17 types of specific HIV cure-related studies	WTP ranged from 100 to 78%	N.A.
		*Ranging from survey research to clinical studies*	Autologous transplants, efficacy studies and blood draws all had 100%. Latency reversing agents had the lowest WTP with 78%	

*PWHIV* people with HIV, *WTP* willingness to participate, *ATI* analytical treatment interruption, *N.A.* not applicable, because it was not reported

The majority of the survey research that measured hypothetical WTP also identified several individual characteristics that influenced hypothetical WTP. Older PWHIV were hypothetically less willing to participate [33, 34, 38], as were women [35], PWHIV with a lower level of education [34], and minority populations such as African American people and Hispanic people in the US [33, 35]. A multi-country survey found that PWHIV from the UK and US had higher hypothetical WTP compared to PWHIV from other countries [38]. Hypothetical WTP was lower when PWHIV’s financial status was lower [24, 35]. Personal health also played a role in hypothetical WTP. For example, characteristics associated with HIV, such as having an unsuppressed viral load (> 50 copies/ml) and having a history of two or more ART regimens were associated with higher WTP [33], as was lower CD4-count (201–305 cells/µL) [38]. Additionally, PWHIV with a longer time since diagnosis, who did not have a sexually transmitted infection in the past year, and PWHIV who perceived themselves as ‘very healthy’ had lower hypothetical WTP [24, 35, 40], as did PWHIV with limited knowledge of HIV cure and HIV treatment [38]. PWHIV who had no previous research experience [45] and perceived cure research to be safe due to trial monitoring were hypothetically more willing to participate. Similarly, PWHIV who perceived cure research as not burdensome, low risk [35, 41], and as having high benefit [33, 35], also had higher hypothetical WTP. Higher hypothetical WTP was also associated with taking advice from healthcare professionals [40], autonomous decision-making [41], and positive affective evaluations in informed consent forms [26]. Furthermore, self-identification as an HIV activist and self-confidence as PWHIV were associated with higher hypothetical WTP [36].

A large number of both qualitative and quantitative studies described *barriers and facilitators* to hypothetical WTP (Supplementary Table 5). We distinguished three barriers to hypothetical WTP. The first was potential *clinical and medical risks*. Perceived risks included side effects, fear of physical pain or uncomfortable procedures, potential ART resistance, permanent harm, and even death [22, 23, 25, 29–32, 35, 38–40, 42–44, 46–52]. The second barrier was possible *social risks*, such as concern about transmitting HIV while participating in clinical trials, being treated poorly by the research staff, and privacy concerns [22, 23, 25, 30, 31, 35, 41–43, 46, 48, 49, 51–56]. Lastly, some barriers were rooted in *practical considerations* such as fear that research participation would interfere with day-to-day life, needing to take time off from work or family, financial risks, and health insurance concerns [23, 29, 30, 35, 39, 42, 44, 46, 48–53, 55, 57, 58].

In addition to the three barriers, we also distinguished four *facilitators* for PWHIV’s hypothetical WTP. The facilitator most often reported was *altruism* [23, 27–32, 34, 39, 42, 43, 45–52, 55, 58–61]. This included PWHIV’s desire to help others and contribute to scientific knowledge. The second facilitator focused on *perceived personal benefits* [23, 28–30, 32, 34, 35, 39, 41–43, 46–52, 55, 56, 58, 60, 61], including possible health and psychological improvements, possible stigma reduction, the acquisition of more knowledge about HIV cure (research), better access to care, and financial compensation. The third facilitator related to *personal experiences and beliefs*. Several studies also showed that PWHIV were motivated by their own experiences with previous HIV (cure) research and HIV treatment (interruptions) and by their personal belief in science [25, 43, 50, 54]. The fourth facilitator pertained to *study conditions* that have the potential to limit risks or barriers to participation [27, 32, 39, 40, 43, 45, 51, 52, 55–58, 62–65]. Studies described how PWHIV desired credible organizations and reliable research staff to ensure good treatment and privacy [32, 40, 43, 54, 55, 57, 58, 63, 65]. To be mindful of potential medical and clinical risks, some studies found that PWHIV wanted close monitoring in clinical trials, especially in analytical treatment interruptions (ATIs) [32, 43, 47, 51, 55, 58, 63, 65]. Frequent viral load and CD4-count testing were particularly valued as this not only monitors participants’ well-being but could also decrease HIV transmission risks during ATIs. Studies concerned with at-home viral load testing observed that most PWHIV responded positively to this possibility [57, 63, 64]. Several studies also suggested access to diverse prevention strategies to reduce the risk of HIV transmission during ATIs [51, 54, 56, 65]. Prevention strategies could include access to information, condoms, and PrEP availability for partners. Lastly, some studies showed that PWHIV indicated that they wanted support for themselves or their partners during clinical trials [43, 45, 51, 54].

Although several facilitators and barriers were identified, some studies argued that the decision to *engage* in HIV cure research was personal and unique for each individual [41, 51, 55, 66, 67]. Generally, qualitative studies described the process by which PWHIV carefully vet information while considering and balancing the risks and benefits of participating in HIV cure research [55, 67]. To aid PWHIV in the decision-making process, several studies suggested that PWHIV prioritized discussing participation in HIV cure research with healthcare providers and loved ones [44, 45, 47, 55, 61, 67]. Most studies that evaluated decision-making processes in HIV cure research argued that participants required sufficient information to make reasonable decisions [45, 47, 55, 58, 59]. This is also supported by diverse studies that found that most PWHIV who participated in HIV cure research had a strong understanding of the study, made rational choices, and were satisfied with their decision [47, 58, 60, 61, 68].

A few studies reported on PWHIV’s *experiences* of participating in early-phase HIV cure clinical trials. Given the early phases of research, studies often reported results based on small samples. Research found that the majority of participants in HIV cure clinical research had positive experiences and believed to have benefited from participating [47, 57, 68, 69]. Benefits reported included furthering HIV cure research and contributing to the HIV community. Perceived personal benefits were related to better access to HIV care as well as HIV (cure) knowledge [57, 60, 61, 69]. In line with these observations, a few studies also reported that most participants were willing to participate in similar studies or would recommend participation to other PWHIV [57, 69]. This was attributed to experienced benefits, positive interactions with research staff, and lower anticipated physical burden [57, 69]. It should also be noted that while most studies found that PWHIV reported a lower anticipated physical burden in ATI trials [57, 69], the mental burden was often underestimated [69]. This was associated with feelings of uncertainty and loss of control as well as elevated anxiety [57, 58, 61, 63, 69]. Furthermore, two studies, assessing shorter-term treatment interruptions, found that the few HIV cure research participants that participated in these studies did not find it difficult to use measures to prevent HIV transmission during ATI trials [57, 69].

#### Perspectives of Key Populations

The perspectives of key populations (e.g., men who have sex with men, people who inject drugs, transgender people, next of kin, community members, and HIV activists) were most often considered when discussing ATI or end-of-life HIV cure research. Key populations accurately understood the *meaning* and importance of ATIs and end-of-life HIV cure research [22, 53, 70, 71].

A few cross-sectional survey studies showed that people without HIV had lower *WTP* than PWHIV [33, 36]. Nevertheless, we found that *facilitators* of and *barriers* to participation in HIV cure research for key populations were similar to the facilitators and barriers observed for PWHIV. This was reflected by the results of a cross-sectional survey study conducted among both HIV-negative transgender individuals and transgender individuals with HIV, where almost none of the identified risks or benefits to participation were statistically significant [39]. Furthermore, several qualitative studies reported that key populations were also concerned with the possible declining health of partners and HIV transmission risks during ATIs as they identified access to robust partner protection and frequent testing as essential facilitators for ATI research [22, 57, 65]. Research showed that according to partners and other community members, robust partner protection includes various strategies such as counseling, the availability of condoms, PrEP, and frequent viral load testing [22, 57, 65] Two qualitative studies among next of kin showed that they also identified altruism and perceived personal benefits as facilitators for participation for their loved ones in an end-of-life study [71, 72]. Next of kin were proud of their loved one’s altruism and also perceived the end-of-life study as a possibility to navigate the death and grieving process of their loved ones [71]. Barriers were lower in end-of-life research, as their loved ones did not fear physical risks. Concerns for next of kin were the possible interference with palliative care and that participants’ dignity would not remain intact [71, 72].

Multiple key populations did report the importance of *engaging* the community and communicating about HIV cure research [53, 54, 65, 73–77]. In qualitative studies, partners of PWHIV specifically expressed the desire to be actively involved in ATI studies. However, although the disclosure of PWHIV’s participation in HIV cure research was desired by most [22, 54], community members also explained that disclosure was not always possible due to complicated relationship dynamics and psychosocial and cultural factors [53, 54]. Furthermore, a qualitative study showed that community members in the United States highlighted the importance of diversity in engagement as they advocated that more effort should be made to include underrepresented groups, especially those who are disproportionately affected, such as transgender individuals, cisgender women, and racial and ethnic minorities [47]. An effective way to engage key populations was reported in two uncontrolled quasi-experimental studies from South Africa. They found that online interactive educational tools were appropriate communication strategies to increase HIV cure knowledge among PWHIV and their next of kin [78, 79]. Another approach to community engagement discussed in the literature was crowdsourcing contests [74, 75, 77].

#### Perspectives of Professionals

The research among professionals reflected their opinions on several different types of HIV cure research including, ATI, end-of-life research, cell and gene therapy, and combination strategies. Several qualitative and quantitative studies found that most professionals are interested in HIV cure research [28, 80, 81], and find their own role important because they have been involved in creating and evaluating study procedures [66], and because their support is needed for the success of HIV cure research [32]. Furthermore, two cross-sectional surveys among health care providers in France and an interview study among HIV clinicians in the United States indicate that most healthcare providers were supportive of HIV cure trials [28, 81, 82]. However, one cross-sectional survey study among physicians in France showed that when self-reported support was compared with a reluctance score, support for HIV cure research was overestimated [82]. This was also reflected in a qualitative study conducted in the United States where clinical researchers, policymakers, and bioethicists described their hesitance about HIV cure clinical trials that require participants to undergo treatment interruptions [43]. Across both qualitative and quantitative studies, these studies showed that professionals understand HIV cure clinical research, especially ATIs, as risky, with limited benefits, as some believed no direct health or indirect psychological benefits could be guaranteed [43, 48, 49, 83, 84]. The literature also showed that professionals often highlighted physical risks, such as side effects, HIV mutations, and HIV drug resistance, [32, 43, 48, 56, 59, 66, 70, 80, 81, 83–86] and possible unknown risks [32, 43, 66, 70, 80, 85]. Psychosocial risks, such as unrealistic trial expectations, negative impacts on participants’ social life and mental health, privacy concerns, and HIV transmission to sexual partners were also identified by professionals [32, 43, 56, 59, 66, 70, 81, 84, 86].

The perceived physical and psychosocial risks by professionals reflect *barriers* to conducting HIV cure research. To *facilitate* HIV cure research, qualitative research with professionals explored opinions on possible risk reduction, especially regarding ATIs. These studies found that professionals identified several potential strategies to reduce known and unknown physical health risks. These included: only conducting the first-in-human studies with a strong scientific rationale, strict exclusion criteria for participants, and adequate monitoring of participants [62, 70, 86, 87]. The qualitative studies on possible ATI risk reduction also provided suggestions as to how potential psychosocial risks might be limited. Specifically, studies showed that professionals believed that community engagement in HIV cure research could prevent misconceptions and false hope [49, 53, 54, 59, 70, 86, 88]. Studies examining professional opinions on informed consent forms concluded that the term cure should be avoided in informed consent forms and that more effort should be made to improve vague and noninformative language about risks and benefits [49, 86, 89]. Research also found that professionals proposed that, in addition to the physical well-being, the mental well-being of participants should be monitored during trials to ensure that participants’ mental health does not deter nor influence their decisions during trials [27, 62, 70, 90]. Studies also reported professional perceptions regarding prevention strategies for HIV transmission. These included counseling participants on transmission risks, offering a range of prevention measures such as condoms and PrEP for sex partners, and involving partners in HIV cure research [54, 70].

A study reporting the results of a cross-sectional survey of professionals’ *experiences* with HIV cure research described the difficulties of enrolling participants in studies. Potential HIV cure participants were found to be reluctant to undergo invasive procedures that had no benefits and required them to adhere to strict participant inclusion criteria [83]. Moreover, in an interview study, professionals from the United States elaborated on the difficulty of including underrepresented minority groups in HIV cure research [53]. To improve potential participants’ understanding and ultimately participation, professionals highlighted the importance of sustained *community engagement* in HIV cure research across several qualitative studies [53, 54, 70]. Another interview study also reported the importance of engaging healthcare workers as clinicians in the United States and pointed out that their knowledge of local trials and ease of referral could increase support for HIV cure research [81].

### Perspectives on HIV Cure

#### Perspectives of People with HIV

Several studies reported on HIV cure *awareness.* A cross-sectional survey among Brazilian transgender women found that most transgender women with HIV had heard about HIV cure [39]. Research from the United States found that PWHIV knew that an HIV cure was not yet available to them [29, 35]. However, awareness of the details of HIV cure developments seemed to be limited, as an interview study in the Netherlands showed [42]. Similarly, a study conducted in Hong Kong found that less than half of the participants were aware of the concept of a functional cure [40]. Research also reported that most PWHIV wanted to know more about HIV cure [39].

Media reports were found to often feature the term HIV cure prominently, without explaining what a cure *meant* [91]. Research among PWHIV showed that HIV cure was most often understood as an eradicating cure, with most PWHIV associating HIV cure with statements such as not having HIV inside the body and not transmitting HIV [15, 29, 34, 35, 38, 46, 47, 68, 92–94] (Table 3). Several qualitative and quantitative studies from the United States, Australia, and the Netherlands, specifically reported that HIV eradication was preferred over HIV suppression [68, 92–95]. Generally, PWHIV believed that suppression offers less certainty as HIV might resurface [93, 95]. Contrastingly, one Ghanaian cross-sectional survey study recorded that most PWHIV would prefer HIV suppression over HIV eradication, as they placed high importance on continued doctors’ visits [34].

**Table 3:** Stakeholders’ perception of an HIV cure: meaning, impact and stance on HIV cure (N = 26)

Reference	Stakeholders (N)^a^	Meaning of HIV cure	Impact of HIV cure	Stance on an HIV cure^b^
Bonney, Lamptey [34]	PWHIV (N = 282)	Preferred undetectable virus with continued doctor visits and medication (89%); over a detectable virus, but no medication and doctor visits (69%) and complete cure with no doctor visits and no medication (11%)		
Chu, Wu [96]	PWHIV (N = 29)		Inspire hope, decrease fatalistic attitudes, and may reduce stigma	(+) Possibility of being cured renewed hope for regaining physical well-being and social mobility ( −) Some also believed that they will not have access to a cure even if it were available
	P (N = 12)		Increase testing uptake and linkage to care. It could increase incidences of STIs	(=) Cure might not be necessary, because of the success of ART
Dube, Eskaf [46]	PWHIV (N = 282)	The most desired were not having HIV inside the body, No more transmission	No longer taking ART	
Dube, Evans [35]	PWHIV (N = 400)	Not transmitting HIV, eliminating HIV from the body	No more treatment	(+) Only 3% thought a cure would never materialize
Dube, Hosey [47]^c^	PWHIV (N = 29)	*Survey*: having HIV eliminated from the body	*Open question*: Freedom, hope, empowerment, life, and living without fear. Putting away stigma *Survey*: being able to stop with HIV medication, not having to think about HIV, having no future health concerns related to HIV, a better quality of life	
Dubé, Simoni [44]	PWHIV (N = 19)			(=) Did not want to take risky interventions that would jeopardize the effectiveness of the current treatment
Fridman, Ubel [92]	PWHIV (N = 454)	Participants favored eradication over suppression		
Kwan, Chan [40]	PWHIV (N = 356)	Explores the concept of functional cure	Restoration and stabilization of effective immune function (63%), not being able to transmit HIV (53%), lowered risk of AIDS or related morbidity (especially among treatment-naïve 46% in comparison with treatment-experienced 35%), reduced clinic visits (more among treatment-experienced 34% than treatment-naïve 18%)	(+) Most participants anticipated an HIV functional cure
Lau, Smith [28]	PWHIV (N = 442)			(+) 55% thought a cure would be achievable in 10 years
	P (N = 144)			(+) 19% thought a cure would be achievable in 10 years
Lewin, Attoye [99]	P (N = 542)	Optimal target: HIV elimination. Minimum target: HIV was at least 2 years under effective virus control (suppression)		
Ma, Wu [97]	PWHIV (N = 22)		Improved health, hope, and happiness	(+) Some believed HIV could be cured ( −) some were suspicious of cure availability and feasibility ( −) Some believed that HIV would never be cured
Mathews, Farley [74]^c^	KP		Improve health and happiness for PWHIV	
McMahon, Elliott [68]	PWHIV (N = 20)	90% rated a ‘sterilizing cure’ as very desirable compared to 55% for the ‘functional cure’	Most important: Not being able to transmit HIV, and being unable to be reinfected with HIV Stopping ART, stopping doctor visits, and being considered as someone not infected with HIV	
Moodley, Rossouw [80]	P (N = 14)			( +) Initial enthusiasm, ( =) but reluctant to temper with the success of ART ( −) A cure out of reach was not a cure at all
Moodley, Staunton [85]^c^	P (N = 9); KP (N = 3); PWHIV (N = 3)		The cure was viewed as a “return to normality” with no need to take medication again	( +) A cure would be great, ( −) but something that would occur in the far future
Murray, Kratka [24]	PWHIV (N = 200)		Reduce future health complications	
Poteat, Aqil [29]	PWHIV (N = 10)	Completely free of HIV	Stop taking daily medications, enhanced sexual intimacy, no longer suffer from depression, and improve their life in general	( +) Optimistic attitude towards HIV cure discovery, which was based on the improvements of HIV medications (-) Some believed that a cure was being hidden from the public ( −) Other participants were pessimistic and if it would be discovered it would be too expensive
Power, Dowsett [95]	PWHIV (N = 20)	Elimination of HIV from the body. HIV suppression was not seen as a cure	It would ensure their future health, remove the everyday worry and be a relief from the burden of stigma	
Power, Westle [30]	PWHIV (N = 20)			( +) Participants were optimistic based on their own emotional experiences ( −) Some actively avoided information as it would create false hope. Others expressed cynicism about commercial motivation
Romijnders, de Groot [94]	PWHIV (N = 29) KP (N = 13)	HIV cure is when HIV is completely removed from the body if there is no chance of rebound of viral load, and if there is no risk of transmitting HIV anymore (elimination) Post-treatment control (suppression) was viewed by participants as the next step in HIV treatment, but not as a cure	PWHIV could be like everybody else again. No need for disclosure Both PWHIV and KP hoped cure would reduce stigma Both PWHIV and KP hoped it would improve sexual freedom	( +) Most PWHIV and KP hoped that a cure would become a reality ( =) Other PWHIV were neither hopeful nor pessimistic and seemed to want to manage expectations ( −) PWHIV who were struggling with living with HIV seemed unable to perceive a cure and its plausibility
Saberi, Eskaf [52]	PWHIV (N = 282)		Younger PWHIV noted that they could plan for a better future, not be required to disclose their status, have no stigma, and no longer think of stigma or dying Elder PWHIV were mainly concerned with spending less on healthcare and worrying about their health	
Simmons, Kall [38]	PWHIV (N = 1703)	No longer having HIV in your body	Most desired outcomes were no risk of HIV-related health problems and never needing to take medications	
Sylla, Evans [93]	PWHIV (N = 76)	Participants described HIV eradication and when asked about suppression many expressed apprehension	No longer taking medication, sexual freedom, and end to stigma	( +) Excited about a sterilizing cure (= /−) apprehension about a functional cure
van Paassen, Dijkstra [42]	PWHIV (N = 20)	Most participants perceived HIV cure as complete elimination, whereas only a few people thought of it as post-treatment control (suppression)	No more medication (long-term damage worries, the burden of daily medication, ART reminds of diagnosis) Impact on relationships (No longer living with a secret, starting a new relationship), becoming normal (starting over, normalization)	( +) Some participants found a cure important as it could improve their quality of life ( =) Some found a cure not so important as ART already feels like a cure
Wozniak, Cerqueira [39]	KP (N = 71)			( =) A minority of participants thought that there is no need for an HIV cure (15%) (-) or that there would never be a cure for HIV (24%) ( +) and almost half (47%) of respondents thought there would be a cure in the future
	PWHIV (N = 47)			( +) 59% believed there to be a cure for HIV ( =) 29% believed that a cure is not needed
Wu, Zhang [98]	PWHIV (N = 26)		The majority was optimistic that a cure would reduce HIV stigma. However, some were a bit more skeptical and pessimistic about the impact of HIV cure on stigma	
Zhang, Pan [15]^c^	PWHIV; KP; P (N = 471)	Believed that a cure would remove all traces of HIV	Decrease HIV stigma/discrimination, it could improve interpersonal relations, and social stability, and change sexual attitudes	

*PWHIV* people with HIV, *KP* key populations, *P* professionals

^a^Stakeholders include: PWHIV; key populations, including men who have sex with men, people who inject drugs, transgender people, next of kin, community members, and HIV activists; professionals such as researchers, healthcare professionals, social workers, policymakers, and pharmaceutical industry representatives

^b^Three stances on HIV cure: positive (+), negative (−), and in favor of status quo (=)

^c^Perceptions of the different individual stakeholder groups were not distinguishable

In the literature on PWHIV’s perspectives on HIV cure, one focus group study, conducted in the United States, found that the term sterilizing cure, used to describe the complete elimination of HIV from the body, is considered problematic by some PWHIV. That is because the term has negative connotations with sterility or forced sterilization [93]. That same study reported that PWHIV often associate the term functional cure with the term remission. Although the term remission can be useful because of its familiarity, many perceived the term to be intimidating because it is often used in relation to cancer, and cancer is associated with recurrence and death [93]. Another experimental study among PWHIV from the United States concluded that it was important to correctly describe HIV eradication or suppression, irrespective of the term used [92].

Across several qualitative and quantitative studies conducted in various countries (Table 3), we distinguished three *stances* on an HIV cure held by PWHIV: positive stance, negative stance, and stance which favored the status quo. The most often reported stance was positive and optimistic [28–30, 35, 39, 40, 42, 85, 93, 94, 96, 97]. Research showed that most PWHIV believed an HIV cure was achievable and associated the *impact* of HIV cure with concepts such as hope, freedom, empowerment, improved health, a normal life, happiness, and an overall better quality of life [15, 24, 29, 35, 38, 40, 42, 46, 47, 52, 68, 84, 93–95, 97, 98]. Some examples of possible perceived impacts were: no longer needing ART, fewer HIV care providers’ visits, fewer health concerns, and lower healthcare costs. Additionally, PWHIV outlined positive impacts that went beyond their health and healthcare needs describing how a cure could potentially lead to closer interpersonal relationships, enhanced sexual intimacy, improved mental health, and reduced HIV stigma. Furthermore, optimistic attitudes seemed to be based on the improvements in HIV treatment medication [29], and people’s experiences living with HIV [30, 94, 97], rather than factual knowledge about HIV cure [30].

The second stance also found across several qualitative and quantitative studies was negative [29, 30, 85, 93, 94, 96]. A study reporting the results of focus group discussions among black transgender women living with HIV in the United States found that several participants were pessimistic or frustrated about the discovery of an HIV cure as they had a limited understanding of the challenges to finding an HIV cure [29]. Two other studies also found that some PWHIV did not believe a cure would be developed [30, 39], positing commercial interest in ART as the reason for this [30]. Furthermore, qualitative studies from China and the United States showed that PWHIV from more socio-economic disadvantaged backgrounds believe that, if a cure were to become available, it would not be accessible to them due to financial or structural barriers [29, 96, 97]. Additionally, several studies, reporting both qualitative and quantitative findings, found that some PWHIV were pessimistic about the impact of HIV cure because they expected little to no changes. The stigma around HIV would remain due to its association with stigmatized behavior and marginalized groups, and the quality of life would not significantly improve for those with access to ART [39, 42, 44, 94, 96–98]. The third stance was one of favoring the status quo. Interview studies from the United States and the Netherlands, and cross-section research from Brazil showed that several PWHIV were satisfied with their current treatment and did not need an HIV cure, as they believed that the prospect of an HIV cure would not be worth the risk [39, 42, 44, 94].

#### Perspectives of Key Populations

Few papers addressed key populations’ views on HIV cure [15, 39, 74, 75, 85]. Three papers showed through crowdsourcing contests in China and the United States that key populations perceived the *meaning* of an HIV cure to be an eradicating cure [15, 74, 75]. Additionally, these crowdsourcing studies and one interview study from South Africa showed that most key populations believed that an HIV cure could reduce stigma, improve relationships, cause more social stability, and change sexual behavior [15, 74, 75, 85]. Furthermore, similar to PWHIV, *stances* on HIV cure could be distinguished into three categories. Some key populations were pessimistic or believed that a cure was not necessary, but most were optimistic and believed that there would be a cure in the future [39, 85] (Table 3).

#### Perspectives of Professionals

While it is likely that most professionals were generally recruited for their knowledge and expertise about the field of HIV cure research, the level of awareness among professionals was explicitly assessed in one study. This study found that about half of the interviewed healthcare professionals in South Africa had heard about HIV cure [85]. In that study, professionals also stated that they did not know what an HIV cure would mean as, currently, there is no cure. A Delphi study among multiple professionals from various countries reported a high level of agreement with the ultimate goal of an HIV cure, namely the “complete eradication of the rebound-competent reservoir in a safe, effective, and scalable manner, with the cured individual being protected for life from reinfection” [99], p. 49]. Although more challenging, in this same study a consensus was also reached on a minimum definition of HIV cure, namely “individuals’ plasma HIV RNA [should be] below the level at which transmission occurs […] The cure should provide an individual with at least 2 years of effective virus control” [99], p. 46]. In this minimum definition, protection from reinfection was not required [99].

Compared to PWHIV, professionals’ *stances* on HIV cure were not as optimistic. In a survey conducted in France, 55% of the PWHIV surveyed, but only 19% of the healthcare professionals surveyed, believed there would be a cure in the next 10 years [28]. Similarly, in two qualitative studies, various professionals in South Africa perceived HIV cure as something that would only occur in the distant future. They were hesitant to tamper with the success of ART as an HIV cure was perceived as risky [80, 85]. Likewise, physicians in China believed that a cure might not be necessary because ART, which they already describe as a kind of cure, is successful. Moreover, while the Chinese physicians believed a cure may positively *impact* linkage to care, they noted it may also lead to an increase in risky behavior [96].

## Discussion

Social science research in the field of HIV cure research is growing, with reports on the perspectives of over twelve thousand stakeholders to date and the number of papers published increasing in recent years. The importance of social science has also been acknowledged by the IAS Global Cure Strategy as it has the potential to complement basic science and empower stakeholders through social engagement [11]. To consolidate the current social science literature in the field of HIV cure research and specifically, stakeholder engagement, this systematic review set out to provide an overview of the perspectives of various HIV cure stakeholders.

The two main themes in the literature were: stakeholder perceptions on HIV cure research and stakeholder perceptions on HIV cure. Our analysis of stakeholder perceptions on HIV cure research indicated that the hypothetical level of WTP in HIV cure research was relatively high. However, we also found that actual levels of WTP were lower among PWHIV and professionals. The reviewed research also identified associated (individual) characteristics of hypothetical WTP, as well as facilitators to and barriers of hypothetical HIV cure research. The reviewed research established that the majority of stakeholders regarded clinical HIV cure research as risky. Clinical and social risks were identified by all stakeholders. Nevertheless, studies showed that PWHIV and key populations were motivated to participate in hypothetical HIV cure research by altruism, perceived personal benefits, and personal experiences and beliefs. Contrastingly, studies also reported that professionals found it more difficult to identify facilitators as no benefits could be guaranteed. Instead, professionals were found to focus more on identifying facilitators that could minimize risks. Several clinical and psychosocial risk management strategies were identified across the included studies. The reviewed research also considered the perspectives of PWHIV and key populations in risk management strategies. Rather than clinical risk management, we observed that studies focused more on the prevention of psychosocial risks. Our review demonstrated that PWHIV, key populations, and professionals suggested similar social risk prevention strategies such as the availability of transmission prevention for partners, guidance, and comprehensive research protocols. The findings of this review related to the second main theme of stakeholder perceptions on HIV cure showed that most stakeholders were optimistic about HIV cure. Our findings showed that most stakeholders defined HIV cure as eradicating and outlined positive associated impacts such as more freedom, potential stigma reduction, changes in sexual behaviors, and increases in testing and linkage to care. However, our review also observed that compared to PWHIV and key populations, professionals were less optimistic and more accepting of HIV suppression as a form of cure.

The IAS Global Cure Strategy recommends that research in the field of HIV cure seeks to understand how characteristics and social considerations influence participation in HIV cure research [11]. Our review addressed this in part by reporting on stakeholders’ (hypothetical) WTP in HIV cure research, identifying associated characteristics, and outlining barriers and facilitators to participation in HIV cure research. These results were comparable to observations in the related fields of HIV prevention and cancer research. HIV prevention research has also demonstrated that hypothetical WTP in HIV vaccine trials was high [100, 101], but actual WTP in HIV vaccine trials was lower [102]. Additionally, our review has shown that motivations for participation in HIV cure research are relatively similar to motivations for participation in HIV vaccine trials [102], and cancer trials [103–106].

While the included papers addressing these themes often had strong conceptual rationales and lenses guided by the principles of meaningful involvement of PWHIV, community engagement, or research ethics principles, we found that the use of behavioral theories, which provide insight into the proximal factors that may shape behavior and may mediate broader social and structural influences, was limited. Based on these observations, and in accordance with IAS Global Cure Strategy’s suggestions for more theoretically engaged research [11], we recommend that future research seeking to better understand stakeholders’ decision-making processes with regard to engagement and participation in HIV cure research examine behavioral theories addressing factors that shape people’s decisions [107]. As in existing research, further theoretical inspiration could be drawn from related fields. For example, research could be informed by conceptual approaches used to assess decision-making processes related to PrEP, condom use, or sustained ART use, as well as regarding participation in clinical cancer trials.

Given the large number of theories of behavior and behavioral change, various theories can be considered to help understand how characteristics and social considerations influence participation in HIV cure research [108]. One theory to consider is the Theoretical Domains Framework (TDF). The TDF combines 128 explanatory constructs from 33 behavioral theories and provides a framework of theoretical domains to explain barriers and facilitators of behavior in any particular situation [108]. While the TDF combines several important explanatory constructs from 33 behavioral theories, it does not specify relationships between the theoretical domains and constructs [108]. Therefore, other theories such as the Necessity-Concerns Framework [109, 110] could also provide promising insights for stakeholder engagement in HIV cure research. Following this model, decisions to engage in HIV cure research would be based on stakeholders’ perceived needs and experienced concerns regarding an HIV cure. People’s perceived need for an HIV cure is shaped by their HIV-related illness perceptions [111], which reflect their ideas about the symptoms associated with HIV, the cause, duration, and consequences of HIV infection, as well as the possibility to control or recover from HIV [111]. People’s experienced concerns about an HIV cure are shaped by their treatment perceptions [109], which comprise general beliefs about medicines and specific beliefs about the effectiveness and adverse impacts of potential HIV curative strategies [109]. The Necessity-Concerns Framework has proven value in HIV treatment research [110], with indicators of people’s perceived necessity and experienced concerns regarding ART found to be critical covariates of sustained ART use [112], and ART adherence [113].

The IAS Global Cure Strategy has also included the recommendation that stakeholder perceptions should guide the development of an HIV cure [11]. The reviewed research shows that most stakeholders described HIV cure as HIV eradication from the body. While challenging, a Delphi study reached a consensus on the minimum attributes that would make an HIV cure acceptable among professionals. Yet, it is not fully apparent what the minimum attributes that would make an HIV cure acceptable are among PWHIV and key populations. The difficulty in reaching a consensus among professionals and the lack of clarity among PWHIV and key populations about the minimum attributes for an HIV cure might be explained by the current state of science. Various potential strategies, such as latency reversal, latency silencing, gene therapy, vaccines, antibodies, and immunotherapy, are still under investigation, making it difficult to predict what a future HIV cure would look like. It could be especially difficult for PWHIV and key populations to predict what a future HIV cure would look like, as several studies found that awareness of developments in the field of HIV cure research was limited. The exact level of knowledge of the developments in the field of HIV cure among PWHIV and key populations remains unclear. Some studies among PWHIV and key populations reported minimal awareness of the developments of HIV cure research in general, while others reported limited knowledge of specific details. Accordingly, we recommend that future research further explore PWHIV and key populations’ awareness of HIV cure to better comprehend their understanding of HIV cure and to assess which attributes of an HIV cure are important to them. Moreover, to ascertain from which minimum point an HIV cure would be considered acceptable, we also suggest that research is conducted among diverse stakeholders in various contexts as preferred attributes of HIV cure may vary based on individual characteristics, location, or experiences.

The IAS Global Cure Strategy has also identified various ethical issues associated with the development of an HIV cure as research priorities [11]. Like the IAS Global Cure Strategy, this review observed that ethical concerns regarding ATIs have been well explored. However, results of current studies about possible HIV transmission prevention measures used during ATIs are equivocal, with some stakeholders reporting no challenges and others reporting difficulties adhering to prevention measures. Also, there is limited research on HIV transmission prevention measures for studies assessing longer periods of treatment interruptions. Moreover, while existing research reports on numerous potential strategies to reduce the risk of ATIs, recent studies found that the emotional impacts of ATIs were underestimated. We hence recommend that potential behavioral, psychosocial, and emotional risk reduction strategies during ATIs should be explored further, including for longer ATIs. This review also found that ethical issues regarding end-of-life studies were discussed. We found that these studies were carefully designed, and both PWHIV and key populations had mostly positive associations with study participation. Recent research has also begun to explore ethical issues with respect to cell and gene therapy or combination HIV cure strategies. However, this was much less addressed, which may reflect that these cure research strategies are not as concrete. Therefore, we suggest further research into stakeholder perspectives on cell and gene therapy and combination HIV cure strategies.

Furthermore, while diverse stakeholders have already been engaged by previous research, we propose that future studies on stakeholder perspectives and engagement in HIV cure actively seek to diversify the representation of stakeholders even more and focus on other stakeholders in addition to PWHIV and on settings beyond the Global North. In our review, we found that the majority of studies assessed the perspectives of PWHIV (N = 66, 84.62%); fewer studies assessed the perspectives of key populations (N = 23, 29.49%) and professionals (N = 31, 39.74%). This discrepancy should be remedied. In addition, most studies were conducted with stakeholders in the Global North (N = 56, 71.79%) and, more specifically, in the US (N = 39, 50.00%). Future research should endeavor to investigate the perspectives of stakeholders around the world, in particular in low- and middle-income countries most affected by HIV.

Moreover, from our review of the literature, it became apparent that stakeholder engagement was important and valued. To engage stakeholders, diverse methodologies were used, such as community advisory boards, interviews, surveys, focus groups, Delphi methods, discrete choice experiments, deliberative methods, crowdsourcing, and dyadic analyses. Like other appraisals of stakeholder engagement [114], we also noted that HIV cure stakeholders were predominantly engaged in early phases and less in later stages of research. Involvement in the first stage of research (i.e., research question development) was well reported in numerous studies with HIV cure research acceptability. Similarly, the involvement of stakeholders in protocol design was reported by several studies addressing ethical issues and required study conditions. Stakeholder opinions were also considered in participant recruitment and enrollment, in particular in research assessing attitudes about trial participation and opinions on the informed consent process. Moreover, a recent and growing body of research addresses stakeholder views on participant enrollment and follow-up, including motivations, experiences, and retention in actual HIV cure trials. In contrast, stakeholder engagement in the data analysis, interpretation of findings, drawing of conclusions, developing of recommendations, and dissemination stages were rarely reported. We recommend that stakeholder engagement in the field of HIV cure research continues to support and empower stakeholders in the earlier stages of research and also develop strategies to engage stakeholders in the later stages. Additionally, to ensure meaningful and robust social engagement in the field of HIV cure research, we recommend the inclusion of diverse and triangulated methodologies.

### Limitations

This systematic review should be interpreted in light of some limitations. First, the varying definitions of HIV cure (research) limited the comparability of data. We attempted to overcome this by adopting an all-inclusive approach and by providing details on specific definitions where appropriate. Second, the large number of studies from the Global North and, specifically, the US, may limit the geographical generalizability of the findings. We recognize this and have highlighted a need to document the views of stakeholders from all over the world. A final possible limitation is that we classified different individual stakeholders for the sake of parsimony. However, the stakeholders in each group are heterogeneous and their perspectives may, likewise, be heterogeneous. As a result, where possible, we described the individual stakeholder groups in the results.

## Conclusion

This review has synthesized the perspectives of PWHIV, key populations, and professionals on the field of HIV cure research. We recommend that future research include an even greater diversity of stakeholders and incorporate theories of behavior such that we can explore how stakeholders decide to meaningfully engage in HIV cure research. We also recommend more research on stakeholders’ awareness and opinions of different HIV cure strategies, including cell and gene therapy and combination strategies. Lastly, we recommend that various avenues for communicating and social engagement with HIV cure stakeholders in all phases of research are explored.

### Supplementary Information

Supplementary File 1 (DOCX 51 KB)

## Data Availability

All data is available.
