# Supplementary material for: The Importance of Social Engagement in the Development of an HIV Cure: A Systematic Review of Stakeholder Perspectives
Source: AIDS Behav. 2023 Jun 17;27(11):3789–812. doi: 10.1007/s10461-023-04095-z (PMC10589186; doi:10.1007/s10461-023-04095-z)
Supplement: Supplementary file 1 — (DOCX 51 KB) [file 10461_2023_4095_MOESM1_ESM.docx]

**SUPPLEMENTARY MATERIAL**

**Supplementary Table 1.**

*Search terms*

| **Population:**  **stakeholders of HIV cure** |  | **Investigated condition:**  **HIV cure (research)** |  | **Outcome:**  **Stakeholder views** |
| --- | --- | --- | --- | --- |
| “People Living with HIV” OR  “Persons living with HIV” OR  PLHIV OR  PWHIV OR  PLWHIV OR  PLWH OR  “HIV-positive” OR  “Key populations” OR  “Men who have sex with men” OR  MSM OR  “people who inject drugs” OR  PWID OR  partners OR  stakeholders OR  participant OR  participants OR  volunteers OR | AND | “HIV cure” OR  “HIV cure-related clinical trials” OR  HCRCT OR  “HIV cure-related trials” OR  “analytical treatment interruption” OR  ATI OR  “AIDS Clinical Trials Group” OR  ACTG OR  “HIV remission” OR  “cured of HIV” OR  “HIV research” OR  “Berlin Patient” OR  “Timothy Brown” OR  “London Patient” OR  “Düsseldorf patient” OR  “Mississippi baby” OR  “Boston patients” OR | AND | Perspective OR perception OR experience OR attitude OR importance OR significance OR awareness OR meaning OR stigma OR implication OR ethics OR  ethical OR  risk OR  risks OR  benefit OR benefits OR “Consent Process” OR  willingness OR “decision making” |

**Supplementary Table 2.**

*Characteristics of the individual papers (N = 78)*

| **Reference** | **Location of the study** | **Study design** | **Sample size (N)** | **Type of Stakeholder**† | **Main theme(s)** |
| --- | --- | --- | --- | --- | --- |
| Arnold, Evans (33) | United States | Quantitative | 2094 | PWHIV | HIV cure research |
| Barr and Jefferys (83) | Multi-country | Quantitative | 73 | Professionals | HIV cure research |
| Bonney, Lamptey (34) | Ghana | Quantitative | 251 | PWHIV | HIV cure  HIV cure research |
| Campbell, Dube (22) | United States | Qualitative | 20 | PWHIV  Key populations | HIV cure research |
| Chu, Wu (96) | China | Qualitative | 40 | PWHIV  Professionals | HIV cure |
| De Scheerder, van Bilsen (69) | Belgium | Mixed methods | 11 | PWHIV | HIV cure research |
| Dube, Agarwal (64) | United States | Qualitative | 17 | PWHIV | HIV cure research |
| Dube, Agarwal (65) | United States | Qualitative | 20 | PWHIV Key populations | HIV cure research |
| Dubé, Dee (66) | United States | Qualitative | 36 | PWHIV  Professionals | HIV cure research |
| Dube, Eskaf (87) | United States | Quantitative | 17 | PWHIV | HIV cure research |
| Dube, Eskaf (46) | United States | Quantitative | 282 | PWHIV | HIV cure  HIV cure research |
| Dube, Eskaf (57) | United States | Quantitative | 73 | PWHIV  Key populations  Professionals | HIV cure research |
| Dubé, Evans (43) | United States | Qualitative | 36 | PWHIV  Professionals | HIV cure research |
| Dube, Evans (35) | United States | Quantitative | 400 | PWHIV | HIV cure  HIV cure research |
| Dube, Hosey (47) | United States | Mixed methods | 29 | PWHIV | HIV cure  HIV cure research |
| Dube, Kanazawa (53) | United States | Qualitative | 21 | PWHIV  Key populations  Professionals | HIV cure research |
| Dube, Kanazawa (54) | United States | Qualitative | 21 | PWHIV  Key populations  Professionals | HIV cure research |
| Dube, Kanazawa (62) | United States | Qualitative | 20 | PWHIV  Key populations  Professionals | HIV cure research |
| Dube, Kanazawa (70) | United States | Qualitative | 19 | PWHIV  Key populations  Professionals | HIV cure research |
| Dubé, Kanazawa (63) | United States | Qualitative | 18 | PWHIV  Professionals | HIV cure research |
| Dubé, Patel (72) | United States | Qualitative | 7 | Key populations | HIV cure research |
| Dubé, Simoni (44) | United States | Qualitative | 19 | PWHIV | HIV cure  HIV cure research |
| Dubé, Taylor (48) | United States | Qualitative | 36 | PWHIV  Professionals | HIV cure research |
| Evans (67) | United States | Qualitative | 4 | PWHIV | HIV cure research |
| Fiorentino, Protière (36) | France | Quantitative | 195 | PWHIV | HIV cure research |
| Fridman, Ubel (92) | United States | Quantitative | 454 | PWHIV | HIV cure |
| Gilbertson, Kelly (49) | United States | Qualitative | 26 | PWHIV  Professionals | HIV cure research |
| Gilles, Lesage (45) | Switzerland | Qualitative | 15 | PWHIV | HIV cure research |
| Henderson, Peay (55) | Thailand | Qualitative | 12 | PWHIV | HIV cure research |
| Henderson, Waltz (58) | Thailand | Mixed methods | 8 | PWHIV | HIV cure research |
| Hendricks, Nair (78) | South Africa | Quantitative | 51 | PWHIV  Key populations | HIV cure research |
| Hendricks, Varathan (79) | South Africa | Quantitative | 88 | PWHIV  Key populations | HIV cure research |
| Javadi, Mathur (71) | United States | Qualitative | 7 | Key populations | HIV cure research |
| Julg, Dee (86) | Multi-country | Qualitative | 41 | Professionals | HIV cure research |
| Kanazawa, Gianella (90) | United States | Qualitative | 20 | PWHIV  Professionals | HIV cure research |
| Kanazawa, Gianella (27) | United States | Qualitative | 20 | PWHIV  Professionals | HIV cure research |
| Kratka, Ubel (50) | United States | Qualitative | 22 | PWHIV | HIV cure research |
| Kwan, Chan (40) | Hong Kong | Quantitative | 356 | PWHIV | HIV cure  HIV cure research |
| Lau, Smith (73) | Australia | Qualitative | 40 | PWHIV  Key populations  Professionals | HIV cure research |
| Lau, Smith (28) | Multi-country | Quantitative | 586 | PWHIV  Professionals | HIV cure  HIV cure research |
| Lau, Smith (56) | Multi-country | Quantitative | 586 | PWHIV  Professionals | HIV cure research |
| Lessard, Dube (23) | Canada | Mixed methods | 37 | PWHIV | HIV cure research |
| Lewin, Attoye (99) | Multi-country | Mixed methods | 542 | Professionals | HIV cure |
| Ma, Wu (97) | China | Qualitative | 22 | PWHIV | HIV cure |
| Mathews, Farley (74) | United States | Mixed methods | 72 | Key populations | HIV cure research |
| Mathews, Farley (75) | United States | Qualitative | 144 | Key populations | HIV cure research |
| McMahon, Elliott (68) | Australia | Quantitative | 20 | PWHIV | HIV cure  HIV cure research |
| Moodley, Rossouw (80) | South Africa | Qualitative | 14 | Key populations  Professionals | HIV cure  HIV cure research |
| Moodley, Staunton (85) | South Africa | Qualitative | 15 | PWHIV  Key populations  Professionals | HIV cure  HIV cure research |
| Moodley, Staunton (89) | South Africa | Qualitative | 68 | PWHIV  Key populations  Professionals | HIV cure research |
| Murray, Kratka (24) | United States | Quantitative | 200 | PWHIV | HIV cure  HIV cure research |
| Neergaard, Jones (61) | United States | Qualitative | 15 | PWHIV | HIV cure research |
| Peay, Ormsby (59) | Thailand | Qualitative | 12 | PWHIV  Key populations  Professionals | HIV cure research |
| Peay, Rennie (41) | Thailand | Quantitative | 408 | PWHIV | HIV cure research |
| Perry, Dube (60) | United States | Qualitative | 13 | PWHIV | HIV cure research |
| Perry, Taylor (88) | United States | Qualitative | 15 | Professionals | HIV cure research |
| Poteat, Aqil (29) | United States | Qualitative | 10 | PWHIV | HIV cure  HIV cure research |
| Power, Dowsett (95) | Australia | Qualitative | 20 | PWHIV | HIV cure |
| Power, Fileborn (91) | Australia | Qualitative | 95 | N/A‡ | HIV cure |
| Power, Westle (30) | Australia | Qualitative | 20 | PWHIV | HIV cure  HIV cure research |
| Prakash, Gianella (37) | United States | Quantitative | 473 | PWHIV  Key populations | HIV cure research |
| Preau, Doumergue (25) | France | Qualitative | 51 | PWHIV  Professionals | HIV cure research |
| Prins, Paulus (31) | Netherlands | Quantitative | 477 | PWHIV | HIV cure research |
| Protiere, Arnold (84) | France | Quantitative | 358 | PWHIV  Professionals | HIV cure research |
| Protiere, Fressard (82) | France | Quantitative | 164 | Professionals | HIV cure research |
| Protière, Spire (32) | France | Mixed methods | 82 | PWHIV  Professionals | HIV cure research |
| Romijnders, de Groot (94) | Netherlands | Qualitative | 42 | PWHIV  Key populations | HIV cure |
| Saberi, Campbell (51) | United States | Qualitative | 20 | PWHIV | HIV cure research |
| Saberi, Eskaf (52) | United States | Quantitative | 282 | PWHIV | HIV cure |
| Sauceda, Dubé (26) | United States | Quantitative | 86 | PWHIV | HIV cure research |
| Simmons, Kall (38) | Multi-country | Quantitative | 1703 | PWHIV | HIV cure  HIV cure research |
| Sylla, Evans (93) | United States | Qualitative | 76 | PWHIV | HIV cure |
| Sylla, Patel (81) | United States | Qualitative | 12 | Professionals |  |
| van Paassen, Dijkstra (42) | Netherlands | Qualitative | 20 | PWHIV | HIV cure  HIV cure research |
| Wozniak, Cerqueira (39) | Brazil | Quantitative | 118 | PWHIV  Key populations | HIV cure  HIV cure research |
| Wu, Zhang (98) | China | Qualitative | 26 | PWHIV | HIV cure |
| Zhang, Pan (15) | China | Qualitative | 471 | PWHIV  Key populations  Professionals | HIV cure |
| Zhao, Day (77) | China | Qualitative | 31 | PWHIV  Key populations  Professionals | HIV cure research |

Abbreviations: PWHIV, People with HIV; N/A: not applicable

†Stakeholders include: PWHIV; key populations, including men who have sex with men, people who inject drugs, transgender people, next of kin, community members, and HIV activists; experts such as researchers, healthcare professionals, social workers, policy makers, and pharmaceutical industry representatives

‡Type of stakeholder not applicable because study included content analysis of newspapers or informed consent forms

**Supplementary Table 3.**

*The National Institute for Health and Clinical Excellence Quality Appraisal Score (N = 78)*

| **Reference** | **Quantitative score†** |  | **Qualitative score‡** |
| --- | --- | --- | --- |
|  | **Internal validity** | **External validity** | **Overall assessment** |
| Arnold, Evans (33) | + | + |  |
| Barr and Jefferys (83) | - | + |  |
| Bonney, Lamptey (34) | + | + |  |
| Campbell, Dube (22) |  |  | + |
| Chu, Wu (96) |  |  | + |
| De Scheerder, van Bilsen (69) § | + | - | + |
| Dube, Agarwal (64) |  |  | + |
| Dube, Agarwal (65) |  |  | + |
| Dubé, Dee (66) |  |  | + |
| Dube, Eskaf (87) § | + | - | + |
| Dube, Eskaf (46) | + | _+_ |  |
| Dube, Eskaf (57) | + | + |  |
| Dubé, Evans (43) |  |  | + |
| Dube, Evans (35) | + | + |  |
| Dube, Hosey (47) § | + | - | + |
| Dube, Kanazawa (53) |  |  | + |
| Dube, Kanazawa (54) |  |  | + |
| Dube, Kanazawa (62) |  |  | + |
| Dube, Kanazawa (70) |  |  | + |
| Dubé, Kanazawa (63) |  |  | + |
| Dubé, Patel (72) |  |  | + |
| Dubé, Simoni (44) |  |  | + |
| Dubé, Taylor (48) |  |  | + |
| Evans (67) |  |  | - |
| Fiorentino, Protière (36) | + | + |  |
| Fridman, Ubel (92) | ++ | + |  |
| Gilbertson, Kelly (49) |  |  | + |
| Gilles, Lesage (45) |  |  | + |
| Henderson, Peay (55) |  |  | + |
| Henderson, Waltz (58) § | + | + | + |
| Hendricks, Nair (78) | + | + |  |
| Hendricks, Varathan (79) | + | + |  |
| Javadi, Mathur (71) |  |  | + |
| Julg, Dee (86) |  |  | + |
| Kanazawa, Gianella (90) |  |  | + |
| Kanazawa, Gianella (27) |  |  | + |
| Kratka, Ubel (50) |  |  | + |
| Kwan, Chan (40) | + | + |  |
| Lau, Smith (73) |  |  | + |
| Lau, Smith (28) | + | + |  |
| Lau, Smith (56) | ++ | + |  |
| Lessard, Dube (23) § | + | + | + |
| Lewin, Attoye (99) § | + | + | + |
| Ma, Wu (97) |  |  | + |
| Mathews, Farley (74) § | + | + | + |
| Mathews, Farley (75) |  |  | + |
| McMahon, Elliott (68) | + | + |  |
| Moodley, Rossouw (80) |  |  | + |
| Moodley, Staunton (85) |  |  | + |
| Moodley, Staunton (89) |  |  | + |
| Murray, Kratka (24) | + | + |  |
| Neergaard, Jones (61) |  |  | + |
| Peay, Ormsby (59) |  |  | + |
| Peay, Rennie (41) | + | + |  |
| Perry, Dube (60) |  |  | + |
| Perry, Taylor (88) |  |  | + |
| Poteat, Aqil (29) |  |  | + |
| Power, Dowsett (95) |  |  | + |
| Power, Fileborn (91) |  |  | + |
| Power, Westle (30) |  |  | + |
| Prakash, Gianella (37) | + | + |  |
| Preau, Doumergue (25) |  |  | + |
| Prins, Paulus (31) | + | + |  |
| Protiere, Arnold (84) | ++ | + |  |
| Protiere, Fressard (82) | ++ | + |  |
| Protière, Spire (32) § | + | + | + |
| Romijnders, de Groot (94) |  |  | + |
| Saberi, Campbell (51) |  |  | + |
| Saberi, Eskaf (52) | + | + |  |
| Sauceda, Dubé (26) | ++ | + |  |
| Simmons, Kall (38) | ++ | ++ |  |
| Sylla, Evans (93) |  |  | + |
| Sylla, Patel (81) |  |  | + |
| van Paassen, Dijkstra (42) |  |  | + |
| Wozniak, Cerqueira (39) | + | + |  |
| Wu, Zhang (98) |  |  | + |
| Zhang, Pan (15) |  |  | + |
| Zhao, Day (77) |  |  | + |

†Quantitative papers received a methodological quality rating for internal and external validity. The score ranged from a high score (++), when all or most of the criteria items had been fulfilled. A medium score (+), when some of the checklist criteria have been fulfilled. Or a low score (-), when few or no checklist criteria have been fulfilled and the conclusions were likely to alter
‡ Qualitative papers received an overall assessment. The score ranged from a high score (++) when all or most of the criteria items had been fulfilled. A medium score (+), when some of the checklist criteria have been fulfilled. Or a low score (-), when few or no checklist criteria have been fulfilled and the conclusions were likely to alter
§Mixed method papers received a score for both quantitative internal and external validity as well as an overall qualitative assessment. The score ranged from a high score (++) when all or most of the criteria items had been fulfilled. A medium score (+), when some of the checklist criteria have been fulfilled. Or a low score (-), when few or no checklist criteria have been fulfilled and the conclusions were likely to alter

**Supplementary Table 4.**

*Summary of themes identified through thematic synthesis*

| **Main theme** | |
| --- | --- |
| Sub-theme | **Explanation** |
| **Perspectives on HIV cure research** | Research directed toward an HIV cure encompasses various social, behavioral, and ethical aspects. This theme deals with stakeholders’ perspectives related to the research process of HIV cure. |
| Meaning of HIV cure research | Stakeholders’ understanding/definition of HIV cure research and their assigned value of HIV cure research |
| Willingness to participate | Stakeholders’ hypothetical or actual willingness to participate or support HIV cure research. This sub-theme also describes individual characteristics associated with hypothetical or actual willingness to participate |
| Facilitators and barriers of  HIV cure research  participation | Stakeholders’ facilitators and barriers for participation in or support of HIV cure research. Barriers deal with various identified risks and practical considerations. Facilitators include several motivations and benefits as well as strategies to manage the identified risks. |
| Engagement of stakeholders  in HIV cure research | Stakeholders’ engagement in HIV cure research, community involvement, and important ethical aspects of engaging stakeholders. |
| Experiences of HIV cure  research | Stakeholders’ experiences of participating or conducting HIV cure research. |
| **Perspectives on HIV cure** | Research concerned with stakeholders’ perspectives on and understanding of HIV cure is included in this sub-theme. |
| Awareness of HIV cure | Stakeholders’ knowledge about (the developments of) an HIV cure. |
| Meaning of HIV cure | Stakeholders’ understanding or definition of an HIV cure. Stakeholders’ opinions of the concepts of an HIV eradication versus an HIV suppression. |
| Impact of HIV cure | The impact of an HIV cure on the future of stakeholders |
| Stance on HIV cure | Stakeholders’ stance (positive, negative, neutral) on an HIV cure. |

**Supplementary Table 5.**

*Facilitators and Barriers for participation in HIV cure research of PWHIV (n = 35)*

| **Reference** | **Facilitator** | **Barrier** |
| --- | --- | --- |
| Bonney, Lamptey (34) | Altruism  Personal benefits: health improvement, gaining knowledge, financial compensation |  |
| Campbell, Dube (22) |  | Clinical/medical risks: viral load resistance, infections, death  Social risk: transmitting HIV |
| Dube, Agarwal (65) | Altruism  Personal benefits: financial compensation  Personal beliefs: confidence in science  Study conditions: regulatory requirements of clinical research, robust partner protection, reducing heteronormative biases | Clinical/medical risks  Social risk: worries about transmitting HIV  Study conditions: access to the study site |
| Dube, Eskaf (46) | Altruism: helping other PWHIV and contributing to research.  Personal benefits: direct clinical benefits, financial compensation | Clinical/medical risks: physical pain, developing ART-resistant virus, significant change to one’s immune system.  Social risks: treated poorly by the research staff, risk of transmitting HIV  Practical concerns: losing health insurance |
| Dube, Eskaf (57) | Study condition: home-based viral load monitoring, good patient-carer relationship | Practical concerns: worried about accuracy/mistakes of home-based viral load monitor. Some preferred clinics. |
| Dubé, Evans (43) | Altruism: helping to find a cure  Personal benefits: financial compensation  Study conditions: standard protocol in place, minimize burden related to frequent monitoring visits, adequate support for participants.  Personal experiences and beliefs: positive experience with previous treatment (interruption) | Clinical/medical risk: going unknowingly to being detectable, risk of developing resistance to ART, increased opportunistic infections  Social risk: transmitting HIV |
| Dube, Evans (35) | Personal benefits: gaining knowledge, health improvement | Clinical/medical risks: developing cancer, resistance to drugs, invasive study procedure, unable to predict viral rebound, side effects.  Social risks: transmitting HIV, discrimination, stigma, being recognized as PWHIV, losing “HIV-positive identity”    Practical concerns: Time away from work, time away from family, transport, time commitment |
| Dube, Hosey (47) | Altruism: helping find a cure and ensuring representation of women  Personal benefits: the desire to be cured, health benefits, access to medical care, psychological benefits, stigma improvement, and compensation. | Clinical/medical risks: possible side effects |
| Dube, Kanazawa (53) | Personal beliefs: trust in biomedical science (mistrust especially in racial and ethnic minorities) | Social risks: disclosure, partners of women (greater stigma)  Practical concerns: environment unwelcoming to women |
| Dube, Kanazawa (54) | Study conditions: clear informed consent forms, support during ATIs, HIV transmission prevention toolbox, PrEP for partners, mental health counseling | Social risks: HIV transmission |
| Dubé, Kanazawa (63) | Study conditions: close monitoring (perhaps home-based viral load testing), clear criteria for restarting ART, approval of HIV care provider |  |
| Dubé, Simoni (44) |  | Clinical/medical risks: undiscovered consequences, side effects, fear of becoming paralyzed or incapacitated.  Practical concerns: interference with daily life. |
| Dubé, Taylor (48) | Altruism: advancing scientific knowledge  Personal benefits: psychological benefits, gaining knowledge | Clinical/medical risks: resistance to ART, cancer, pain, permanent harm, death.  Social risk: poor treatment by research staff, transmitting HIV, disclosure, unwanted media attention, stigma  Practical concerns: Financial risks, losing access to loved ones |
| Evans (67) | Altruism: desire to help others and contribute to a world that has a cure for HIV |  |
| Gilbertson, Kelly (49) | Altruism  Personal benefits: psychological benefits, indirect healthcare benefits, gaining knowledge, intimate relationship benefits, positive behavioral changes, and financial benefits. | Clinical/medical risks: cancer-related risks, drug side effects  Social risk: disclosure during research  Practical concerns: interference with daily life  Transportation, time commitment. |
| Gilles, Lesage (45) | Altruism: advancing science, contributing like others before them  Study conditions: patient-physician relationship |  |
| Henderson, Peay (55) | Altruism: scientific advancements and creating benefits for society, aid the research team  Personal benefits: stopping ART  Study conditions: trust in the organization and research staff, and close monitoring. | Social risk: Privacy concerns  Practical concerns: burden of time and travel. |
| Henderson, Waltz (58) | Altruism: advancing scientific research and helping others  Personal benefits: the opportunity to stop ATI  Study conditions: promised weekly screenings, and trust in research staff. | Practical concerns: the time commitment required was too much. |
| Kanazawa, Gianella (27) | Altruism: contribution to research  Study conditions: community engagement |  |
| Kwan, Chan (40) | Study conditions: therapy safety, advice from healthcare professionals, and the credibility of institutions were the top three factors. | Clinical/medical risks: CD4 count going down, viral load going up and becoming infectious again, concerns about returning to ART |
| Kratka, Ubel (50) | Altruism: sacrifice yourself for the community  Personal benefits: psychosocial benefit, avoiding future health changes, nothing to lose from a change in the current situation.  Personal experiences and beliefs: positive attitude towards healthcare research | Clinical/medical risks: unwilling to risk death  Practical concerns: negative attitude towards healthcare research, a cure is not worth it, uncertainty about taking risks |
| Lau, Smith (28) | Altruism: benefitting others and advancing HIV cure research  Personal benefits: benefitting myself |  |
| Lau, Smith (56) | Personal benefits: financial compensation  Study conditions: home-based viral load testing and home visits of nurses, and partners receiving PrEP. | Social risks: Transmitting HIV |
| Lessard, Dube (23) | Altruistic benefits: benefitting science and society, giving back  Personal benefits: the opportunity to learn more | Clinical/medical risk: permanent/irreversible damage, physical discomfort  Social risk: disclosure during research  Practical concerns: control over body, commutes to the hospital, complex procedures, time-consuming, reduce quality of life, research interferes with participants’ will |
| Peay, Rennie (41) | Altruism  Personal benefits: experimental intervention would boost the immune system. | Social: Transmitting HIV |
| Perry, Dube (60) | Altruism: nested within the context of community, scientific advancement, and moral obligation  Personal benefits: emotional/psychological, financial benefits |  |
| Poteat, Aqil (29) | Altruism: Findings a cure to be around for their children, improve the health of the transgender community, and build social cohesion among transgender women.  Personal benefits: directly improve participants’ health. | Clinical/medical risks: death, sickness, side effects, not being able to return to ART  Practical concerns: not wanting to jeopardize ART success, uncertainty about researchers’ competency, uncertainty about what would be happening, and logistical factors such as time and travel. |
| Power, Westle (30) | Altruism: Opportunity to give something back to the community or future generations  Personal benefit: only one participant referred to the possibility of being cured | Clinical/medical risks: risk of becoming unwell.  Social risks: HIV transmission  Practical concerns: not being able to work, losing control of HIV by stopping treatment. |
| Preau, Doumergue (25) | Personal experiences and beliefs: Previous ART treatment advances shape opinions. | Clinical/medical risks: fears of HIV mutations and resistance  Social risks: HIV transmission |
| Prins, Paulus (31) | Altruism: contributing to scientific knowledge. | Clinical/medical risks: viral rebound, invasive procedures  Social risks: HIV transmission |
| Protière, Spire (32) | Altruism: ensuring medical advances for future generations  Personal benefits: gaining knowledge, one day forgetting about the disease, financial compensation  Study condition: patient’s physician also needed to agree with the trial and preferably also supervise them during the trial. Participants needed to be closely monitored | Clinical/medical risks: irreversible side effects |
| Saberi, Campbell (51) | Altruism  Personal benefits: stigma reduction, elimination of HIV clinical burdens  Study conditions: Most participants had concerns about ATIs, and indicated that they would want more frequent laboratory testing and protection for their sex partners during this time | Clinical/medical risks: potential physical side effects, psychological distress,  Social risk: the possibility of disclosure because of participating,  Practical concerns: The amount of time required to participate. |
| Saberi, Eskaf (52) | Altruistic: feeling good to contribute to research, helping future PWHIV.  Personal benefits: financial compensation, being treated as a special, and support from family and friends.  Study conditions: offered a meal at a study site | Clinical/medical risks: developing dementia, lasting physical pain, personal health, resistant  Practical concerns: financial risks  Social risks: Transmitting HIV, risk of arrest, being recognized as PWHIV |
| Simmons, Kall (38) |  | Clinical/medical risks: becoming unwell, CD4 count might decrease, and viral load might become detectable. |
| van Paassen, Dijkstra (42) | Altruism: contribution to science  Personal benefits: earlier access to new medication, the possibility of being cured by the trial, temporary no more daily medication | Clinical/medical risks: long-term damage, risk of previous ART not working anymore, risks of an acute retroviral syndrome, becoming detectable, and living with uncertain/unknown risks  Social: Transmission to partners, disclosure to partners  Practical concerns: time, not being able to work/study |
| Wozniak, Cerqueira (39) | Altruism: potential for a longer and healthier life for others.  Personal benefits: longer and healthier life for oneself, gaining knowledge, access to care, compensation  Study conditions: meals at a study site | Clinical/medical risks: dermatological side effects musculoskeletal side effects, HIV-related side effects, toxicities/adverse side effects  Practical concerns: psychological side effects, time away from work, long study visits, staying in the hospital overnight. |

Abbreviations: PWHIV, people with HIV; ART, antiretroviral therapy; PrEP, Pre-exposure prophylaxis; ATI, analytical treatment interruption.
